# Exercise and Horticultural Programs for Older Adults with Depressive Symptoms and Memory Problems: A Randomized Controlled Trial

**DOI:** 10.3390/jcm9010099

**Published:** 2019-12-30

**Authors:** Hyuma Makizako, Kota Tsutsumimoto, Takehiko Doi, Keitaro Makino, Sho Nakakubo, Teresa Liu-Ambrose, Hiroyuki Shimada

**Affiliations:** 1 Department of Physical Therapy, School of Health Sciences, Faculty of Medicine, Kagoshima University, Kagoshima 890-8544, Japan; 2 Department of Preventive Gerontology, Center for Gerontology and Social Science, National Center for Geriatrics and Gerontology, Aichi 474-8511, Japan; k-tsutsu@ncgg.go.jp (K.T.); take-d@ncgg.go.jp (T.D.); kmakino@ncgg.go.jp (K.M.); sho-n@ncgg.go.jp (S.N.);; 3 Aging, Mobility, and Cognitive Neuroscience Lab, Department of Physical Therapy, Faculty of Medicine, University of British Columbia, Vancouver, BC V6T 1Z4, Canada; teresa.ambrose@ubc.ca; 4 Center for Hip Health and Mobility, Vancouver Coastal Health Research Institute, University of British Columbia, Vancouver, BC V6T 1Z4, Canada; 5 Djavad Mowafaghian Centre for Brain Health, University of British Columbia, Vancouver, BC V6T 1Z4, Canada

**Keywords:** exercise, horticulture, memory, depression, aging

## Abstract

Depressive symptoms and memory problems are risk factors for dementia. Exercise can reduce these in older people, and horticultural activity can benefit people with dementia. This study assessed the efficacy of exercise and horticultural interventions for community-dwelling older adults with depressive symptoms and mild memory decline. In this randomized controlled trial, older adults (*n* = 89) were assigned to exercise, horticultural, or control groups. Exercise and horticultural programs included 20 weekly 90-min sessions. The control group attended two 90-min classes. Outcomes were assessed at baseline, and then 6- and 12-months post-intervention. Primary outcome measures were the Geriatric Depression Scale-15 (GDS-15) and Wechsler Memory Scale-Revised. Walking speed, two-minute walking test scores, social network, life space, and subjective daily physical activity were secondary outcome measures. Compared with the control group, the exercise group obtained higher immediate and delayed recall logical memory scores, and the increase in immediate recall scores remained 12-months post-intervention. Two-minute walking performance improved in the exercise group, but not after 12 months. GDS-15 scores showed no significant improvements. The horticultural and control groups showed no differences. Exercise may improve memory, while horticultural activity may not. The effects of exercise and horticultural interventions on depressive symptoms remain unclear.

## 1. Introduction

Depressive symptoms and memory problems—both of which are significant risk factors for Alzheimer’s disease (AD)—often co-exist in older adults [1]. Therefore, older adults with depressive symptoms and memory decline should undergo interventions to improve their cognitive functioning and mental health. Exercise is an effective, non-pharmacological intervention for reducing depressive symptoms and improving cognitive functioning in older adults. Physical exercise can also increase hippocampal volume in both cognitively healthy community-dwelling older adults [2] and those with mild cognitive impairment (MCI) [3]. Hippocampal volume is the primary determinant of memory decline [4], and geriatric depression magnifies hippocampal atrophy and the risk of AD [5]. Notably, maintained hippocampal volumes could help reduce risk factors for depressive symptoms among community-dwelling older adults [6]. Therefore, exercise is a useful intervention not only for physical health, but also for cognitive and mental health in older populations.

Horticultural activity is another non-pharmacological intervention strategy for reducing depression. It entails physical activity of moderate intensity, which can promote mental well-being through increased social and behavioral activation in nature-based environments [7]. However, to our knowledge, no well-designed intervention study has examined the effects of horticultural activity on cognitive and mental health (e.g., depressive symptoms, cognitive functioning, and brain volume) in adults at risk of developing dementia. Further, no evidence exists on the effects of exercise on people who are vulnerable to developing both depressive symptoms and memory problems.

We hypothesized that depressive symptoms, cognitive functioning, and brain volume among older adults at a high risk of developing depression and cognitive impairment would improve through physical exercise and participation in horticultural activity. We proposed a 20-week randomized controlled trial (RCT) and a 12-month follow-up assessment to compare the efficacy of physical exercise and horticultural activity versus a control group in community-dwelling older adults with depressive symptoms and mild memory problems.

## 2. Methods

### 2.1. Study Design and Participants

We conducted a community-based, single-blind RCT in Japan. Details of the study protocol have been published [8], and the trial was preregistered (The University Hospital Medical Information Network-Clinical Trials Registry (UMIN-CTR), 000018547). The study population included community-dwelling older adults with depressive symptoms and mild memory decline. Participants had to meet the following inclusion criteria: (1) independent, community-dwelling adults aged ≥ 65 years; (2) presence of depressive symptoms (Geriatric Depression Scale-15 (GDS-15) score of ≥5 [9]); and (3) memory problems (subjective memory complaints or objective mild memory decline indicated by an age-adjusted wordlist memory score at least 1.0 standard deviation (SD) below the reference threshold). The exclusion criteria were as follows: (1) support or care certified by the Japanese public long-term care insurance system; (2) dementia diagnosis or Mini-Mental State Examination (MMSE) score of ≤18; (3) a history of major psychiatric illness (e.g., bipolar disorder) or other serious neurological or musculoskeletal diagnoses; (4) disability in basic activities of daily living; (5) inability to take cognitive performance tests; (6) physical exercise contraindication; and (7) use of walking aids in daily life. Written informed consent was obtained from all participants prior to their enrollment. The study protocol was approved by the Ethics Committee of the National Center for Geriatrics and Gerontology in Japan (#839).

### 2.2. Randomization and Masking

Potential participants in this RCT were recruited from our community-based cohort study (conducted since 2011), which includes face-to-face interviews and measures of physical and cognitive functioning, in Obu city, a suburb of Nagoya, Japan. We identified eligible individuals through a health check survey held between February and October of 2015. Participants were randomly assigned (1:1:1) to an exercise intervention, a horticultural activity intervention, or an educational control group, upon completion of baseline assessments. The randomization sequence was computer generated. A researcher who was unaware of the aims of the study performed the randomization procedure.

### 2.3. Procedures

The three groups were compared at baseline, immediately after intervention (6 months after baseline), and at a 12-month follow-up after intervention completion.

The exercise intervention entailed a multicomponent exercise program consisting of 20 weekly 90-min sessions involving aerobic exercise, muscle strength training, postural balance retraining, and dual-task training. Trained instructors conducted the exercise sessions, which were held at a fitness facility, and approximately 9 to 12 individuals participated in each class. Each session began with a 10-min warm-up period with stretching exercises followed by 20 min of muscle strength exercises and postural balance re-training. The dual-task training exercises were conducted under multitask conditions that included physical and cognitive tasks. We referred to this combined training as “cognicise” (“cogni” for cognition + “cise” for exercise) [10]. For the cognitive tasks in the “cognicise”, multiple domains of cognitive functions, such as working memory, attention, executive function, and language, were included. For example, participants played word games and performed simple calculation tasks while engaging in stepping exercises. In other examples of “cognicise”, participants were instructed to memorize stepping sequences and step exactly according to preplanned forms. The participants also undertook daily home-based exercises and walking, which required self-monitoring using a booklet and a pedometer. The cognicise programs were aimed at preventing the development of dementia [11] and reducing frailty [12].

The horticultural activity program entailed 20 weekly 60- to 90-min sessions involving nature-based group activities. The program included crop-related activities such as cultivating, growing, and harvesting. Individuals in this group engaged in gardening activities including group planting (known as Yoseue-style bonsai), which involved a combination of different plant varieties or shapes and planting flowers in a public garden. Vegetable experts educated the participants on nutritional information and provided recipes for the field crops grown for the purposes of the program.

Participants in the educational control group attended two 90-min education classes during the six-month trial period. The classes included topics (e.g., traffic safety and disaster prevention) that experts considered less likely to influence study outcomes.

### 2.4. Outcome Measures

The primary outcome measures were depressive symptoms and memory performance. The potential reduction in depressive symptoms was determined based on changes in scores on the GDS-15 [13]. Assessments of memory performance included the logical memory subtests of the Wechsler Memory Scale-Revised (WMS-R) [14] and wordlist memory tasks [15]. The WMS-R logical memory subtests in this study used a short story (story A). Participants used headphones to listen to story A, and then were instructed to recall details of the story immediately (immediate recall, 0–25 points) and after 30 min (delayed recall, 0–25 points). The wordlist memory tasks involved immediate recognition and delayed recall of a 10-word target list. Participants were instructed to memorize 10 words, which were presented on a tablet computer. Each target word was presented for two seconds. Thirty words, including 10 target and 20 distraction words, were then presented, and participants were asked to choose the 10 target words immediately; this was repeated for three trials. The average number of correct answers was calculated to produce a total score. Additionally, participants were instructed to recall the 10 target words after approximately 20 min.

Secondary outcome measures included cognitive functions—excluding memory—such as language, attention, executive function, and processing speed. Health-related quality of life (HRQOL), brain volume, and brain-derived neurotrophic factor (BDNF) serum levels were used as secondary outcome measures. Cognitive functioning was assessed using the verbal fluency test (VFT) [16] and tablet versions of the trail-making test (TMT) [15]. HRQOL was assessed using the Short-Form Health Survey-12 (SF-12) [17]. Brain volume data, including whole-brain and hippocampal volume, were evaluated using an Magnetic Resonance Imaging (MRI), a 3-T system (TM Trio, Siemens, Germany). We used the voxel-based specific regional analysis system for Alzheimer’s disease (VSRAD) [18], which enables examination of atrophy of the bilateral medial temporal areas, including the entorhinal cortex (MTA-ERC), using voxel-based morphometry [19]. Serum BDNF levels were measured using the Quantikine Human Kit (R&D Systems, Inc., Minneapolis, MN, USA) [18]. 

Physical performance, social network, life space, and daily physical activity levels were assessed as other outcome measures. Physical performance tests included normal walking speed and a two-minute walking test. Participants’ social networks were assessed using the abbreviated version of the Lubben Social Network Scale (LSNS-6) [20]. Life-space mobility was measured via the life-space assessment (LSA) [21,22]. Daily physical activity levels taken during the two-week period, subsequent to the pre-intervention, post-intervention, and follow-up assessments, were measured using a triaxial accelerometer [23].

Other details of assessment methods for secondary and other outcome measures were described in the protocol [8].

### 2.5. Statistical Analysis

The required sample size for this study was calculated according to our predictions of six-month changes in GDS-15 scores. On the basis of the results of our previous work, conducted in the same town, we predicted that a change of 1.5 GDS-15 points would indicate differences, and we assumed that the SDs for the GSD-15 would be 2.5 in our sample. Assuming a non-consent and dropout rate of 20%–30%, 30 participants were required per group. The study design necessitated a power of at least 80% and an α level of <0.05 to assess the effects of the interventions.

Changes in primary outcome measures, including GDS-15 scores and memory performance post-intervention among participants who completed the intervention and post-assessment immediately following the intervention (six months from baseline), were assessed using a paired *t*-test.

Statistical analyses were conducted to assess the effects of interventions according to the intention-to-treat (ITT) principle using a multiple imputation method for missing data. Between-group differences in primary outcome measures subsequent to the intervention were compared using multiple linear regression analysis. Baseline scores, experimental groups, and characteristics (e.g., age, gender, diagnoses, and medical conditions) were included in the models as covariates. If the effects of the intervention were significant, two planned simple contrasts were performed to assess differences between the exercise and control groups and the horticultural activity and control groups. All analyses were performed using IBM SPSS Statistics version 24.0 (SPSS Inc, Chicago, IL, USA). Statistical significance was set at *p* < 0.05 in all analyses.

## 3. Results

**Figure 1:**
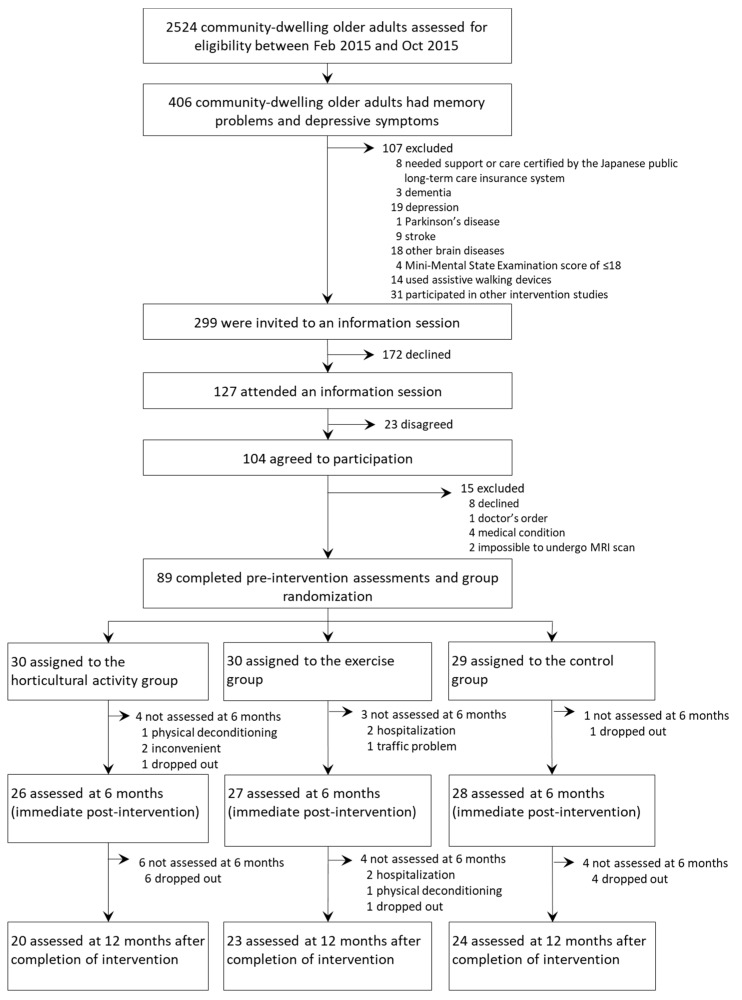
Trial profile.

We assessed 2524 older adults for initial screening between 25 February and 8 October 2015, of whom 406 (16.1%) were identified as potentially eligible participants who had depressive symptoms and memory problems. Of those 406 participants, 112 met the exclusion criteria. Thus, 294 participants who met the inclusion criteria were invited to join our community-based intervention study, and 89 (mean age 73.1 ± 5.5 years, women 50.6%, mean MMSE score 27.7 ± 2.3 points) of those 294 (30.3%) completed pre-intervention assessments and agreed to take part in either the exercise program class (*n* = 30), horticultural activity class (*n* = 30), or educational control group (*n* = 29; Figure 1).

In the exercise group, 27 participants (90.0%, three participants dropped out) completed the post-intervention assessment and 23 (76.7%, seven participants dropped out) completed the 12-month follow-up assessment. In the horticultural activity group, 26 participants (86.7%, four participants dropped out) completed the post-intervention assessment and 20 (66.7%, nine participants dropped out) completed the 12-month follow-up assessment. In the control group, 28 participants (96.6%, one participant dropped out) completed the post-intervention assessment and 24 (82.8%, two participants dropped out) completed the 12-month follow-up assessments. The average participation rate was 91.2% for the exercise group and 84.5% for the horticultural group.

Baseline characteristics of the 89 trial participants were well-balanced across the groups (Table 1). Primary outcomes at baseline did not differ significantly across groups, although TMT-B performance, a secondary outcome measure, significantly differed across groups (Table 2).

**Figure 2:**
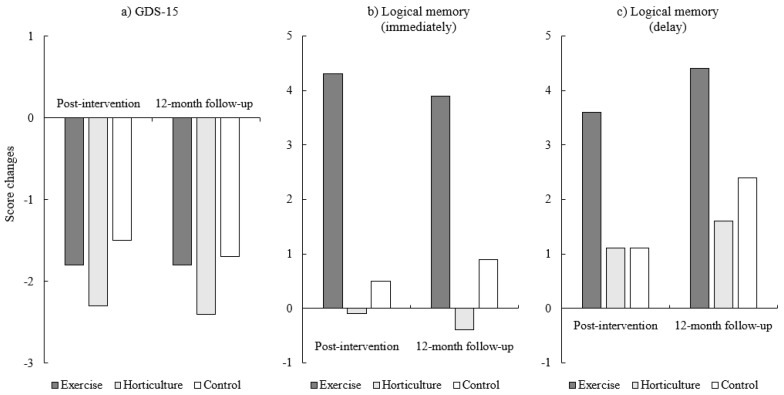
Changes in primary outcomes. GDS = Geriatric Depression Scale.

Changes in primary outcome measures among participants who completed the intervention and assessment immediately following the intervention (six months from baseline) are presented in Table 3. All groups showed significantly decreased GDS-15 scores post-intervention (*p* < 0.01). Performance in logical memory immediate and delayed recall significantly improved only in the exercise group (*p* < 0.01). Although performance in logical memory delayed recall tended to improve in the horticultural activity group, this result was not statistically significant (*p* = 0.09; Figure 2).

Table 4 shows changes since pre-intervention for primary outcome measures including GDS-15 scores, logical memory, and wordlist memory performance. The results of regression analyses, including experimental groups as a covariate for testing between-group differences in GDS-15 scores, indicated no significant between-group differences in post-intervention (*p* = 0.744) and 12-month follow-up (*p* = 0.741) assessments. However, there were significant between-group differences in logical memory immediate (*p* < 0.001) and delayed recall (*p* = 0.007) post-intervention. At the 12-month follow-up, significant between-group differences in logical memory immediate recall (*p* = 0.014) remained, but differences in delayed recall did not (*p* = 0.315). There were no significant between-group differences in wordlist memory performance in the post-intervention (immediate recognition, *p* = 0.333; delayed recall, *p* = 0.883) and 12-month follow-up (immediate recognition, *p* = 0.054; delayed recall, *p* = 0.772) assessments. Planned simple contrasts indicated that participants in the exercise group obtained significantly higher immediate (*p* = 0.001) and delayed logical memory scores (*p* = 0.002) at the post-intervention assessment, and the higher immediate logical memory scores remained at the 12-month follow-up (*p* = 0.017), compared with those in the control group.

The changes from baseline for the secondary outcome measures are presented in Table 5. The results of regression analyses showed no significant between-group differences at the post-intervention and 12-month follow-ups for all secondary outcome measures.

Table 6 shows the changes from baseline for other outcome measures. The results of regression analyses, including experimental groups as a covariate to test between-group differences for the two-minute walking test, indicated significant between-group differences post-intervention (*p* = 0.016). Planned simple contrasts indicated that participants in the exercise group showed significantly improved two-minute walking performance (*p* = 0.011) in the post-intervention assessment, compared with those in the control group. However, no significant improvements in the horticultural activity group were found. There were no significant group differences for changes in walking speed, the two-minute walking test, LSNS-6, or LSA between the pre-intervention and the 12-month follow-up regression analysis results. For daily steps, significant group differences were found at the 12-month follow-up. Planned simple contrasts indicated that, compared with the control group, the horticultural activity group had decreased daily steps at the 12-month follow up.

## 4. Discussion

Participants who completed the intervention and were assessed immediately afterwards (six months since baseline) had significantly decreased GDS−15 scores. However, statistical analyses based on the ITT principle showed that neither exercise nor horticultural activities led to reduced depressive symptoms. Only the exercise intervention showed improved memory functioning effects, and these effects on immediate memory performance remained at the 12-month follow-up assessment. Horticultural activity did not improve memory functioning.

Previous studies have shown physical activity to be associated with a reduced risk of cognitive decline [24] and improved cognitive functioning in older adults [25]. However, an RCT including people with dementia did not reveal any cognitive improvements resulting from an aerobic and strength exercise training program [26]. Therefore, interventions aimed at imparting benefits associated with exercise, including a reduced risk of cognitive decline, must be implemented before the onset of severe cognitive problems (e.g., dementia). Moreover, cognitive training should be implemented alongside physical exercise to improve cognitive functioning. Indeed, a systematic review suggested that combined cognitive and exercise training could improve the cognitive functions and functional status of older adults with and without cognitive impairment [10]. Such interventions may yield cognitive benefits even for older adults with cognitive deficits (e.g., MCI or dementia) [27]. The multicomponent exercise training program in this study included combination training—“cognicise”—requiring cognitive efforts as well as physical exercise. Such a program’s improvement or maintenance of cognitive and physical performance in older adults with MCI has been reported [10]. In our RCT, participants in the exercise group performing multicomponent exercise including cognicise also showed improved memory functioning, and improved effects on immediate memory performance remained at the 12-month post-intervention follow-up. 

Multitasking training may enhance cognitive control in older adults. A previous experimental study showed that older adults who performed multitasking training had cognitive benefits that extended to untrained cognitive control abilities (enhanced sustained attention and working memory) [28]. Our combined exercises and cognitive training included various dimensional cognitive functions (e.g., playing word game, numerical calculation, and memorizing step sequence) during the physical exercise, whereby it is difficult to quantify for cognitive stimulations. However, those multitasking conditions may have driven change in cognitive performance within the exercise groups.

Exercise plays an important role in reducing depressive symptoms. Indeed, a previous meta-analysis showed that, compared with psychological and pharmacological therapies, exercise had a relatively greater effect on depressive symptoms [29]. Another meta-analysis showed that physical exercise had a moderate to large significant effect on depressive symptoms compared with control conditions, but the effect was small and not significant at follow-up [30]. Exercise is recommended as a possible viable adjunct treatment in combination with antidepressants. However, it is unclear whether exercise characteristics (e.g., type, frequency, and duration) influence its effectiveness in reducing depressive symptoms. In the current RCT including older adults with depressive symptoms, exercise did not significantly decrease GDS-15 scores. There was no significant difference in state of depression. It may be that none of the interventions were specifically designed to target the reward system as they do not seem to use an adaptive design that allows them to be in a state of “never too frustrating, never too easy”. Hence, what we see could just be the general effect of behavioral activation. Implementing an adaptive version of “cognicise” or other similar trainings involving multitask could improve its efficacy on depressive symptoms above and beyond control activities.

A previous longitudinal study following 4564 Japanese community-dwelling older adults for an average of 42.6 months suggested that engagement in field work or gardening (hazard ratio 0.71) decreased the risk of dementia [31]. Horticultural therapy may be an effective intervention for mental and behavioral disorders [32].

Possible causes for the lack of significant changes in the horticultural activity group in this study should be discussed. Generally, horticultural activities, including field work and gardening, are considered leisure activities or hobbies. This study adopted an RCT design to maintain a higher evidence level. Therefore, not all participants had prior interest in those activities. In the horticultural activity group, participants took part in horticultural activity classes including growing and harvesting crops once a week. It may have been difficult to continue this activity post-intervention. Conversely, exercise may be easy to engage in as part of one’s daily routine. For example, participants’ progress enhanced their performance of daily home-based muscle strengthening exercises and walking. Therefore, the groups were not equally exposed to the interventions. 

A relatively high rate of wrong cardiovascular conditions (e.g., hypertension and diabetes) was presented. Cardiovascular conditions play an important role in cognition, especially in the executive functions among older adults [33]. More detailed measures of executive functions, such as cognitive control inhibition and set shifting abilities, may be useful for participants with poor cardiovascular conditions. The effects of cardiovascular conditions including medical conditions should have been considered.

Several limitations of this RCT should be noted. Although relatively higher participation rates during the intervention period were found, adherence and continuation rates were not estimated between the completion of the intervention and the 12-month post-intervention follow-up. Drop-out rates immediately following the intervention were around 10% or less in each group; however, drop-out rates at the 12-month follow-up assessment reached approximately 20% to 30%. Also, participants’ motivation to continue the activities practiced in the intervention was not considered. Motivation could affect behavioral changes and continued activities. Additionally, the causes of depressive symptoms were not considered. Interventions to decrease depressive symptoms through exercise among older adults as well as the causes of depressive symptoms, such as physical (e.g., arthritis) [34] and environmental (e.g., living alone) [35] factors should be considered. Future studies and intervention implementations should explore better daily activities and ways of motivating participants to continue daily activities to prevent depressive symptoms and memory problems.

In conclusion, for community-dwelling older adults at a high risk of developing dementia (e.g., with depressive symptoms and memory problems), the community-based group exercise intervention could improve memory. However, the effectiveness of the exercise and horticultural interventions on alleviating depressive symptoms was unclear. Our study provides empirical evidence supporting the effectiveness of community-based, non-pharmacological interventions, which have the potential to reach the general population and assist in maintaining mental and cognitive health.

## Figures and Tables

**Table 1:** Baseline characteristics of the 89 trial participants.

Variable	All (*n* = 89)	Exercise (*n* = 30)	Horticulture (*n* = 30)	Control (*n* = 29)
Age, years	73.1 (5.5)	73.1 (5.3)	73.1 (5.6)	73.0 (5.9)
Female, no. (%)	45 (50.6%)	16 (53.3%)	14 (46.7%)	15 (51.7%)
Height, cm	157.7 (9.3)	158.0 (8.6)	157.4 (8.1)	157.5 (11.3)
Weight, kg	58.4 (11.7)	57.9 (13.4)	61.3 (10.1)	56.0 (11.2)
Education, years	11.8 (2.3)	12.0 (2.1)	11.6 (2.6)	11.7 (2.4)
Medical history, no. (%)				
Hypertension	38 (42.7%)	10 (33.3%)	13 (43.3%)	15 (51.7%)
Diabetes mellitus	22 (24.7%)	8 (26.7%)	7 (23.3%)	7 (24.1%)
Arthritis	24 (27.0%)	8 (26.7%)	10 (33.3%)	6 (20.7%)
Medication, no.	3.3 (3.3)	4.3 (4.5)	3.2 (2.7)	2.3 (1.8)
Grip strength, kg	27.4 (7.9)	26.7 (7.1)	27.8 (7.7)	27.8 (9.1)
MMSE, score	27.7 (2.3)	28.3 (1.7)	27.6 (2.6)	27.1 (2.3)

Data are *n* (%) and mean (SD). no = number; MMSE = Mini-Mental State Examination.

**Table 2:** Comparison of outcome measures in a baseline assessment.

Variable	All (*n* = 89)	Exercise (*n* = 30)	Horticulture (*n* = 30)	Control (*n* = 29)
**Primary outcomes**				
GDS-15, score	6.9 (2.4)	7.0 (2.4)	7.0 (2.4)	6.6 (2.5)
Logical memory (immediately), score	16.8 (6.5)	16.3 (6.9)	16.6 (6.8)	17.6 (5.9)
Logical memory (delay), score	12.0 (6.3)	12.5 (6.3)	11.3 (5.7)	12.1 (7.0)
Word list memory (immediately), score	8.0 (1.4)	8.1 (1.2)	8.1 (1.2)	8.1 (1.5)
Word list memory (delay), score	5.0 (2.1)	5.4 (2.0)	4.3 (2.0)	5.2 (2.2)
**Secondary outcomes**				
Verbal fluency (animal), score	15.3 (4.6)	16.1 (4.3)	14.8 (5.3)	15.0 (4.2)
Verbal fluency (letter), score	20.6 (8.0)	20.6 (7.2)	20.8 (9.0)	20.4 (8.0)
TMT-A, s	20.7 (5.3)	20.2 (5.1)	22.2 (6.4)	19.8 (3.9)
TMT-B, s	40.3 (20.7)	36.5 (21.5)	48.6 (24.4)	36.0 (12.6)
SDST, score	62.7 (11.9)	65.0 (10.8)	58.3 (13.3)	64.7 (10.5)
SF-12, physical health (score), score	46.2 (12.0)	43.5 (15.4)	49.3 (9.6)	45.7 (9.8)
SF-12, mental health (score), score	50.9 (7.1)	49.5 (7.9)	50.8 (7.0)	52.4 (6.2)
SF-12, social health (score), score	47.2 (10.9)	48.4 (12.5)	47.4 (10.2)	45.7 (10.1)
MTA-ERC atrophy, z-score	0.7 (0.5)	0.7 (0.5)	0.7 (0.6)	0.8 (0.4)
WBC atrophy, %	2.0 (1.6)	1.8 (1.3)	2.2 (2.1)	2.0 (1.4)
Serum BDNF level, ng/dL	18.64 (8.35)	18.82 (8.06)	18.01 (8.34)	19.12 (8.87)
**Other outcomes**				
Grip strength, kg	27.4 (7.9)	26.7 (7.1)	27.8 (7.7)	27.8 (9.1)
Walking speed, m/s	1.2 (0.2)	1.2 (0.2)	1.2 (0.2)	1.3 (0.2)
Two-minute walking test, m	149.1 (22.6)	147.1 (19.6)	144.6 (25.8)	155.7 (21.1)
LSNS-6, score	15.3 (5.4)	14.9 (5.8)	16.2 (5.6)	14.8 (4.6)
LSA, score	85.9 (17.4)	80.9 (16.7)	86.6 (17.8)	90.4 (16.9)
Daily steps, steps/day	5569.1 (2614.7)	5311.5 (2460.6)	5795.2 (3235.7)	5601.6 (2060.5)
Moderate physical activity, min/day	48.2 (30.1)	47.2 (31.4)	49.7 (36.2)	47.8 (21.6)

Data are *n* (%) and mean (SD). s = second; m = meter; GDS = Geriatric Depression Scale; TMT = trail-making test; SDST = symbol digit substitution test; SF-12 = Short-Form Health Survey-12; MTA-ERC = bilateral medial temporal areas including the entorhinal cortex; WBC = whole brain cortices; BDNF = brain-derived neurotrophic factor; LSNS = Lubben Social Network Scale; LSA = life-space assessment.

**Table 3:** Changes in primary outcomes for participants who completed the intervention and immediate post-intervention assessments (six months from baseline).

	Exercise (*n* = 27)			Horticultural Activity (*n* = 26)			Control (*n* = 28)		
Primary Outcomes	Baseline	Post-Intervention	*p*	Baseline	Post-Intervention	*p*	Baseline	Post-Intervention	*p*
GDS-15, score	7.1 (2.5)	5.3 (2.5)	**0.002**	6.9 (4.7)	4.7 (2.7)	**<0.001**	6.4 (2.5)	5.1 (3.1)	**0.001**
Logical memory (immediate), score	8.6 (4.3)	12.6 (3.9)	**<0.001**	8.8 (4.3)	8.9 (3.9)	0.836	9.8 (3.3)	10.3 (3.1)	0.417
Logical memory (delay), score	6.4 (3.6)	9.0 (4.0)	**0.001**	5.4 (3.0)	6.4 (3.9)	**0.091**	6.8 (4.2)	7.0 (3.9)	0.638
Word list memory (immediate), score	8.2 (1.1)	8.1 (1.1)	0.548	7.7 (1.5)	7.6 (1.5)	0.604	8.3 (1.4)	8.3 (1.1)	0.944
Word list memory (delay), score	5.7 (1.8)	5.7 (2.0)	1.000	3.8 (1.8)	3.7 (1.9)	0.799	5.5 (1.9)	5.3 (2.3)	0.434

**Table 4:** Mean changes in primary outcome measures.

	Post-Intervention Mean, Mean Change from Pre-Intervention	One-Year Follow-Up Mean, Mean Change from Pre-Intervention
**Exercise**	***n* = 27**	***n* = 23**
GDS-15, score	5.3 (2.5), −1.8 (2.8) ^a^	5.3 (2.9), −1.8 (1.7) ^a^
Logical memory (immediate), score	20.9 (7.0), 4.3 (4.7) ^a^	19.6 (8.8), 3.9 (5.9) ^a^
Logical memory (delay), score	16.7 (6.7), 3.6 (5.2) ^a^	16.3 (7.9), 4.4 (5.0)
Word list memory (immediate), score	8.1 (1.1), −0.1 (0.9)	8.2 (1.1), 0.1 (1.0)
Word list memory (delay), score	5.7 (2.0), 0.0 (1.5)	5.5 (2.1), 0.1 (1.2)
**Horticultural activity**	***n* = 26**	***n* = 20**
GDS-15 (score), score	4.7 (2.7), −2.3 (2.4) ^a^	5.0 (3.5), −2.4 (2.8) ^a^
Logical memory (immediate), score	16.4 (7.0), −0.1 (4.4)	19.2 (6.7), −0.4 (3.5)
Logical memory (delay), score	12.1 (6.4), 1.1 (4.5)	15.7 (7.2), 1.6 (5.1)
Word list memory (immediate), score	7.6 (1.5), −0.1 (0.9)	8.5 (1.2), −0.5 (1.1)
Word list memory (delay), score	3.7 (1.9), −0.1 (1.6)	5.2 (2.2), −0.1 (2.0)
**Control**	***n* = 28**	***n* = 24**
GDS-15 (score), score	5.1 (3.1), –1.5 (2.3) ^a^	4.1 (3.4), −1.7 (2.5) ^a^
Logical memory (immediate), score	18.8 (5.9), 0.5 (5.2)	16.7 (7.9), 0.9 (6.1)
Logical memory (delay), score	13.7 (7.1), 1.1 (4.7)	13.0 (7.4), 2.4 (4.7)
Word list memory (immediate), score	8.3 (1.1), 0.0 (0.9)	6.9 (2.1), 0.1 (0.9)
Word list memory (delay), score	5.3 (2.3), −0.2 (1.2)	3.7 (2.2), −0.2 (1.2)

Data are mean (SD). GDS = Geriatric Depression Scale. ^a^ significantly different from pre-intervention at *p* <0.05.

**Table 5:** Mean changes in secondary outcome measures.

	Post-Intervention Mean, Mean Change from Pre-Intervention	One-Year Follow-Up Mean, Mean Change from Pre-Intervention
**Exercise**	***n* = 27**	***n* = 23**
Verbal fluency (animal), score	16.3 (5.9), −0.2 (4.9)	15.6 (6.3), −1.2 (5.5)
TMT-A, s	19.2 (4.7), −1.1 (3.5)	20.4 (5.7), 0.1 (3.4)
TMT-B, s	33.0 (11.1), −3.2 (20.2)	35.8 (14.9), −0.4 (20.3)
SF-12, physical health (score), score	47.9 (10.3), 5.5 (12.7)	45.3 (14.4), −3.3 (17.3)
SF-12, mental health (score), score	50.3 (7.1), 0.6 (7.5)	49.2 (9.4), 0.3 (9.8)
SF-12, social health (score), score	47.2 (10.3), −0.7 (12.3)	46.4 (10.9), 1.3 (14.3)
MTA-ERC atrophy, z-score	0.6 (0.4), 0.0 (0.1)	0.7 (0.4), 0.1 (0.1)
WBC atrophy, %	1.8 (1.3), 0.0 (0.2)	2.1 (1.6), 0.3 (0.5)
Serum BDNF level, ng/dL	16.03 (8.14), −3.31 (9.62)	18.24 (10.67), −1.32 (13.08)
**Horticultural activity**	***n* = 26**	***n* = 20**
Verbal fluency (animal), score	14.9 (4.9), 0.5 (3.4)	14.2 (6.2), −0.1 (3.3)
TMT-A, s	21.5 (6.1), −1.2 (5.5)	23.4 (8.9), 0.6 (7.7)
TMT-B, s	42.0 (25.1), −8.2 (23.0)	55.4 (63.6), 3.0 (50.9)
SF-12, physical health (score), score	47.6 (9.5), −1.4 (8.4)	45.0 (9.4), 2.2 (9.0)
SF-12, mental health (score), score	51.9 (7.7), 0.4 (10.7)	55.0 (6.2), −2.5 (7.9)
SF-12, social health (score), score	43.7 (13.1), −2.9 (11.0)	42.3 (12.1), 5.6 (10.1)
MTA-ERC atrophy, z-score	0.7 (0.6), 0.0 (0.1)	0.7 (0.7), 0.0 (0.1)
WBC atrophy, %	2.3 (2.2), −0.0 (0.2)	2.6 (2.7), 0.1 (0.3)
Serum BDNF level, ng/dL	15.85 (9.00), −1.14 (11.27)	17.99 (12.15), 1.16 (10.34)
**Control**	***n* = 28**	***n* = 24**
Verbal fluency (animal), score	14.9 (3.8), −0.3 (3.9)	15.2 (3.7), −0.1 (3.2)
TMT-A, s	20.0 (6.4), 0.2 (3.8)	20.4 (7.8), 1.3 (6.3)
TMT-B, s	37.9 (15.4), 3.0 (11.0)	36.1 (15.2), 0.6 (10.1)
SF-12, physical health (score), score	47.4 (9.2), 1.7 (10.4)	47.1 (8.2), −1.8 (10.9)
SF-12, mental health (score), score	50.1 (7.5), −2.1 (7.3)	51.5 (5.8), 0.4 (6.9)
SF-12, social health (score), score	42.6 (13.2), −2.3 (12.3)	43.0 (10.1), 3.5 (10.3)
MTA-ERC atrophy, z-score	0.8 (0.4), 0.0 (0.1)	0.8 (0.3), 0.0 (0.1)
WBC atrophy, %	1.9 (1.0), 0.1 (0.3)	1.8 (1.0), 0.1 (0.3)
Serum BDNF level, ng/dL	14.78 (7.00), −4.04 (11.77)	17.88 (11.68), −0.67 (14.30)

Data are mean (SD). s = second; TMT = trail-making test; SDST = symbol digit substitution test; SF-12 = Short-Form Health Survey-12; MTA-ERC = bilateral medial temporal areas including the entorhinal cortex; WBC = whole brain cortices; BDNF = brain-derived neurotrophic factor.

**Table 6:** Mean changes in other outcome measures.

	Post-Intervention Mean, Mean Change from Pre-Intervention	One-Year Follow-Up Mean, Mean Change from Pre-Intervention
**Exercise**	***n* = 27**	***n* = 23**
Walking speed, m/s	1.3 (0.2), 0.1 (0.2)	1.3 (0.2), 0.1 (0.1)
Two-minute walking test, m	155.8 (23.0), 8.3 (16.1) ^a^	155.6 (23.9), 8.4 (17.3)
LSNS-6, score	14.3 (5.5), −0.6 (3.7)	18.7 (6.3), 3.8 (4.9)
LSA, score	80.4 (12.2), −0.5 (19.2)	82.2 (18.2), 2.7 (19.7)
Daily steps, steps/day	6307.9 (3123.2), 834.1 (2030.5)	6113.4 (3367.8), 583.0 (2379.7)
Moderate physical activity, min/day	54.1 (33.4), 6.6 (18.4)	50.4 (33.6), 2.6 (19.9)
**Horticultural activity**	***n* = 26**	***n* = 20**
Walking speed, m/s	1.2 (0.2), 0.0 (0.1)	1.2 (0.2), 0.0 (0.1)
Two-minute walking test, m	142.4 (23.8), −1.9 (16.6)	145.7 (28.3), 3.5 (16.6)
LSNS-6, score	16.2 (5.2), −0.0 (5.3)	18.1 (6.5), 2.5 (5.7)
LSA, score	83.3 (19.2), −4.4 (15.6)	78.3 (24.3), −9.6 (20.6)
Daily steps, steps/day	5662.6 (2683.6), −132.2 (1677.9)	5018.0 (3218.2), −805.5 (1871.6) ^a^
Moderate physical activity, min/day	45.4 (27.9), −2.8 (20.1)	41.2 (31.2), −7.6 (18.5)
**Control**	***n* = 28**	***n* = 24**
Walking speed, m/s	1.3 (0.2), 0.1 (0.1)	1.3 (0.2), 0.0 (0.1)
Two-minute walking test, m	152.9 (21.5), −3.6 (13.1)	157.5 (22.1), 1.4 (14.1)
LSNS-6, score	14.7 (4.9), 0.4 (3.8)	17.4 (6.1), 2.5 (4.7)
LSA, score	78.9 (16.1), −12.0 (19.5)	81.7 (27.6), −14.4 (29.7)
Daily steps, steps/day	5978.3 (2714.5), 332.7 (2206.5)	5708.8 (2731.1), 229.5 (1989.0)
Moderate physical activity, min/day	56.6 (34.6), 7.5 (30.2)	55.3 (32.3), 8.7 (28.6)

Data are mean (SD). m = meter; LSNS = Lubben Social Network Scale; LSA = life-space assessment. ^a^ significantly different from pre-intervention at *p* < 0.05.
